# Gut Endotoxin Leading to a Decline IN Gonadal function (GELDING) - a novel theory for the development of late onset hypogonadism in obese men

**DOI:** 10.1186/s12610-016-0034-7

**Published:** 2016-06-22

**Authors:** Kelton Tremellen

**Affiliations:** Department of Obstetrics, Gynaecology and Reproductive Medicine, Flinders University, Adelaide, South Australia, Australia

**Keywords:** Male hypogonadism, Testosterone, Endotoxin, Lipopolysaccharide (LPS), Intestinal microbiome, Hypogonadisme masculin, Testostérone, Endotoxine, Lipopolysaccharide (LPS), Microbiome intestinal

## Abstract

Obesity is an increasing public health problem, with two-thirds of the adult population in many Western countries now being either overweight or obese. Male obesity is associated with late onset hypogonadism, a condition characterised by decreased serum testosterone, sperm quality plus diminished fertility and quality of life. In this paper we propose a novel theory underlying the development of obesity related hypogonadism- the GELDING theory (Gut Endotoxin Leading to a Decline IN Gonadal function).

Several observational studies have previously reported an association between obesity related hypogonadism (low testosterone) and systemic inflammation. However, for the first time we postulate that the trans-mucosal passage of bacterial lipopolysaccharide (LPS) from the gut lumen into the circulation is a key inflammatory trigger underlying male hypogonadism. Obesity and a high fat/high calorie diet are both reported to result in changes to gut bacteria and intestinal wall permeability, leading to the passage of bacterial endotoxin (lipopolysaccharide- LPS) from within the gut lumen into the circulation (metabolic endotoxaemia), where it initiates systemic inflammation. Endotoxin is known to reduce testosterone production by the testis, both by direct inhibition of Leydig cell steroidogenic pathways and indirectly by reducing pituitary LH drive, thereby also leading to a decline in sperm production.

In this paper we also highlight the novel evolutionary benefits of the GELDING theory. Testosterone is known to be a powerful immune-suppressive, decreasing a man’s ability to fight infection. Therefore we postulate that the male reproductive axis has evolved the capacity to lower testosterone production during times of infection and resulting endotoxin exposure, decreasing the immunosuppressive influence of testosterone, in turn enhancing the ability to fight infection. While this response is adaptive in times of sepsis, it becomes maladaptive in the setting of “non-infectious” obesity related metabolic endotoxaemia.

## Résumé

L’obésité est un problème de santé publique en expansion ; les deux-tiers de la population adulte de nombreux Pays de l’Ouest sont actuellement soit en surpoids, soit obèses. L’obésité masculine est associée à l’hypogonadisme de survenue tardive, une situation caractérisée par un taux abaissé de testostérone, une qualité spermatique réduite ainsi qu’une diminution de la fertilité et de la qualité de vie. Dans le présent article, nous proposons une nouvelle théorie sous-tendant le développement de l’hypogonadisme lié à l’obésité – la théorie GELDING (Gut Endotoxin Leading to a Decline IN Gonadal function ; Endotoxines intestinales donnant lieu à un déclin de la fonction gonadique).

Plusieurs études observationnelles ont précédemment rapporté une association entre hypogonadisme (testostérone basse) lié à l’obésité et inflammation systémique. Toutefois, nous postulons pour la première fois que le passage trans-muqueuse de lypopolysaccharides bactériens (LPS) de la lumière intestinale dans la circulation constitue un élément inflammatoire clé déclencheur de l’hypogonadisme masculin. L’obésité et une alimentation riche en graisse/riche en calories sont toutes deux signalées pour induire des modifications des bactéries intestinales et de la perméabilité de la paroi intestinale, conduisant au passage d’endotoxines bactériennes (lipopolysaccharide- LPS) de l’intérieur de la lumière intestinale dans la circulation (endotoxémie métabolique) où elles initient une inflammation systémique. Les endotoxines sont connues pour réduire la production de testostérone par les testicules, à la fois par inhibition directe des voies de la stéroïdogenèse des cellules de Leydig et indirectement par la réduction du pic de LH hypophysaire, ce qui conduit aussi à une réduction de la production de spermatozoïdes.

Dans le présent article, nous avons aussi mis en relief nouveaux bénéfices évolutionnaires de la théorie GELDING. La testostérone est connue pour être un puissant immunosuppresseur qui diminue la capacité d’un homme à combattre l’infection. Par conséquent, nous postulons que l’axe reproducteur masculin a élaboré la capacité à diminuer la production de testostérone pendant les périodes d’infection et d’exposition aux endotoxines qui en résulte, ce qui réduit ainsi l’influence immunosuppressive de la testostérone, et en retour augmente la capacité de combattre l’infection. Alors que cette réponse est adaptée en période de sepsis, elle devient inadaptée dans le cadre de l’endotoxémie métabolique ‘non infectieuse’ liée à l’obésité.

## Introduction

### Obesity and its impact on male reproductive health

Obesity has become an increasing public health concern over the last few decades, primarily due to an increase in the availability of calorie dense processed food and the adoption of a sedentary lifestyle. A recent review of body mass index (BMI) changes in 199 countries reported that 20 % of the world population have a BMI above the ideal range (25 kg/m^2^), with a total of 205 million men being obese (BMI ≥ 30 kg/m^2^) [1]. In developed countries such as the United States of America, one-third of the population are known to be overweight and a further third is obese [1]. As obesity is a known risk factor for the development of significant general health concerns such as diabetes, cardiovascular disease, osteoarthritis, poor mental health and early death [2], this increase in obesity is of major public health concern.

In the last decade increasing evidence has also emerged linking obesity with impaired male reproductive health, producing so called late-onset male hypogonadism [3]. Obesity related hypogonadism is characterised by low serum testosterone levels and associated symptoms such as poor libido, erectile dysfunction, depression, lack of motivation, lethargy; as well as somatic symptoms such as muscle weakness, aches and pains [3]. These symptoms can have a major impact on men’s quality of life, especially when they affect relatively young men in their 30’s and 40’s. Furthermore, as testosterone is known to maintain muscle, obesity related hypogonadism produces a decline in muscle mass and therefore basal metabolic rate, meaning that these men burn fewer calories at rest and exercise, predisposing them to gaining further fat - a detrimental positive feedback loop.

Obesity has also been linked with impaired sperm production and function. Recent meta-analyses have linked obesity with a significant increased risk of low sperm count, motility and morphology, plus an increase in sperm DNA fragmentation, resulting in a decline in fertility potential [4, 5]. This decline in sperm quality with increasing BMI may help explain the gradual decrease in the general population’s sperm quality, particularly sperm count, which has been observed to coincide with the increasing trend in obesity over the last few decades [6, 7].

Given the very significant reproductive and general health concerns related to obesity, new approaches to managing this growing epidemic need to be found. While improving diet and increasing exercise are known to reverse weight gain and normalise reproductive hormones [8–10], very few patients are capable of adhering to these lifestyle changes over the long term, resulting in no long term improvements in body composition. However, if it were possible to reverse the decline in serum testosterone associated with obesity, this would not only improve men’s reproductive function and quality of life, but also result in an increase in their lean body mass (muscle) and an increase in their basal metabolic rate [11], producing a sustained reduction in fat mass.

### Current theories behind obesity related male hypogonadism

The current prevailing theory behind obesity related hypogonadism is that the decline in testosterone levels is due to a combination of reduced pituitary LH drive (central hypogonadism) and a direct impairment of testicular function (peripheral hypogonadism) [3, 6]. Adipose tissue contains abundant aromatase activity, an enzyme responsible for the conversion of testosterone to estrogen, with 80 % of male estrogen being derived from the action of aromatase [12]. Therefore, an increase in adipose tissue aromatase results in an increase in the conversion of testosterone to estrogen, reducing serum testosterone levels. Furthermore, estrogen has a “negative feedback” influence on the hypothalamic pituitary (HP) axis resulting in a decrease in anterior pituitary LH pulse frequency and amplitude [3, 6]. Since LH is the prime stimulus for increasing testicular Leydig cell production of testosterone, this estrogen related reduction in LH drive further produces a drop in testosterone production. Blocking aromatase action with letrazole (an aromatase inhibitor) has been reported to result in an increase in LH and testosterone concentration in obese men [13].

White adipose tissue is a major endocrine organ that secretes over 30 biologically active peptides and proteins such as leptin and immunomodulatory cytokines such as TNFα and IL-6 [6]. Under lean conditions leptin increases LH and FSH release by a direct stimulatory effect on the anterior pituitary, and via increasing hypothalamic GnRH pulsatility [14]. However, in obesity leptin levels significantly increase which then results in a functional state of leptin resistance, with impaired leptin action and a resultant decline in HP axis function. Similarly, the pro-inflammatory adipocytokines TNFα and IL-6 have also been reported to impair HP axis function and subsequent testosterone production [6, 15]. Therefore, increased production of estrogen by aromatase, combined with the direct inhibition of the HP axis by leptin and adipose derived inflammatory cytokines, results in a central hypogonadal state.

There is also abundant evidence linking obesity with a direct impairment of testicular function. INSL3, a hormone produced by the Leydig cells independent of pituitary LH drive, has been reported to be negatively associated with BMI, providing evidence for obesity directly impairing Leydig cell function independent of the HP axis [16]. Similarly, levels of inhibin B [17] and AMH [18], both products of the Sertoli cells of the testis, have been reported to decline with increasing BMI, which suggest that obesity also directly impairs Sertoli cell function. Again leptin may play a key role in obesity related testicular dysfunction as leptin has been shown to inhibit the Leydig cell’s production of testosterone [14, 19].

Obesity is known to be characterised in an increase in the production of reactive oxygen species (ROS) and associated oxidative stress, both systemically [20] and within sperm themselves [21]. It is also well established that oxidative stress can impair sperm production and function [22], and there is evidence linking oxidative stress with impaired Leydig cell function [22, 23]. Therefore testicular oxidative stress is also likely to play a significant role in obesity related male hypogonadism.

Finally, morbid obesity is associated with the enveloping of the scrotal contents in pelvic fat tissue, which impedes heat transfer compared to the lean scenario where the testis hang free of the body within the scrotum, maintaining a temperature 2 °C below core body temperature. This adipose related “heating” of the testicles is likely to significantly impair sperm production, since spermatogenesis is optimally performed at 35 °C [24].

## The GELDING theory for obesity related male hypogonadism

### Gelding

*A castrated animal; - usually applied to a horse, but formerly used also of the human male. (Webster’s Dictionary).*

### The GELDING hypothesis

**Fig. 1:**
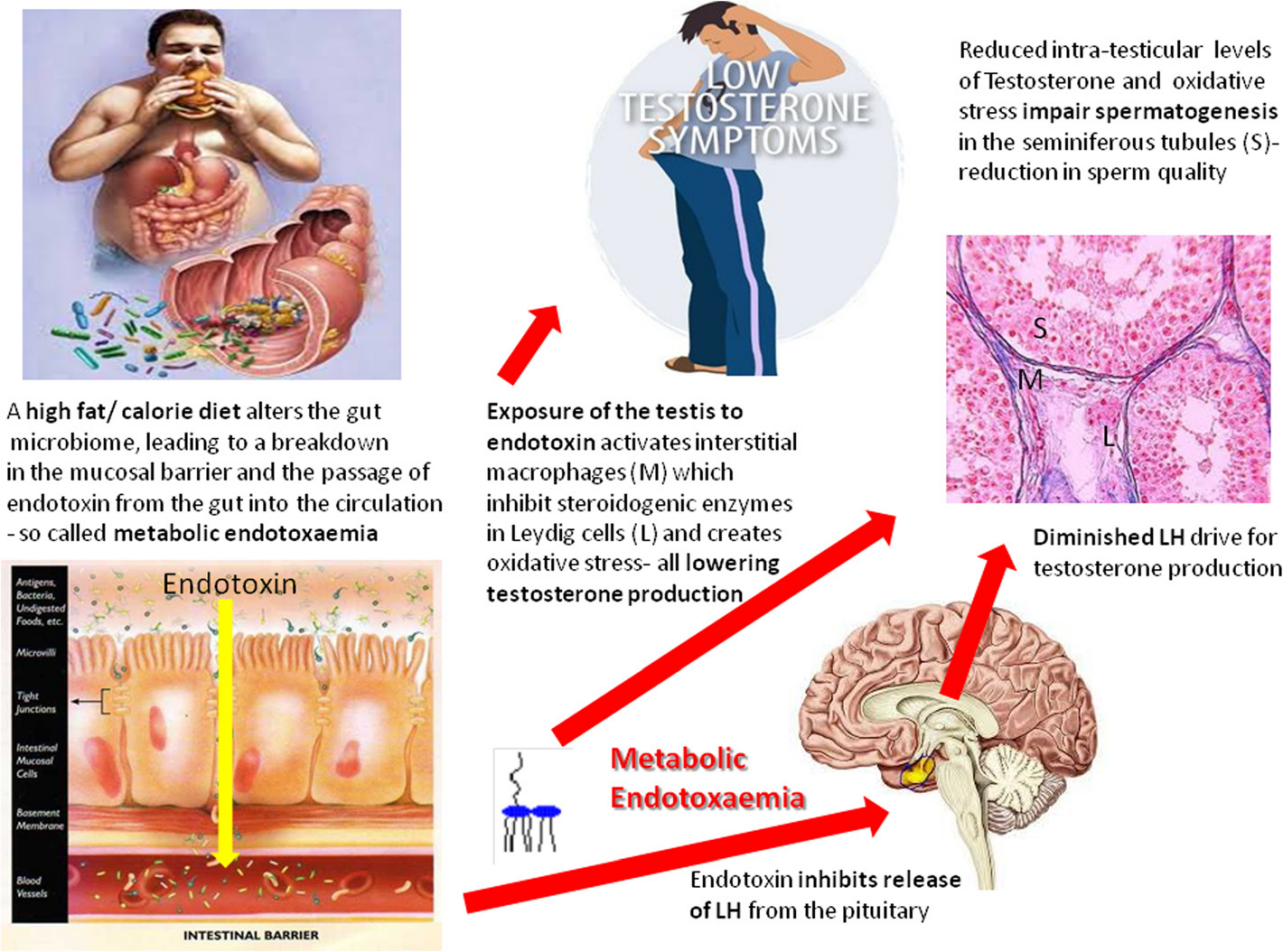
An overview of the GELDING theory of obesity related male hypogonadism

In the GELDING theory of male late onset hypogonadism we hypothesise that obesity, and its associated poor diet (high fat and high calorie), causes a breakdown in the normal intestinal mucosal barrier function (so called “leaky gut”), that then facilitates the passage of gut bacteria from the bowel lumen into the systemic circulation. Here powerful immune stimulants present in bacteria such as endotoxin elicit a chronic state of low grade inflammation (metabolic endotoxaemia) throughout the body that in turn impairs testicular function and reproductive performance (Fig. 1). The GELDING theory of obesity related male hypogonadism is entirely novel, since currently there is no direct evidence linking obesity, metabolic endotoxaemia and impaired testicular function. However, several lines of evidence support the plausibility of this theory.

### Obesity, “leaky gut” and resultant chronic inflammation from metabolic endotoxaemia

The central key to the GELDING theory of male hypogonadism is that activation of the immune system by an obesity related trigger is then capable of impairing testicular function. Several large epidemiological studies have already reported an association between male obesity, markers of inflammation such as CRP and white cell count (WCC), and a reduction in serum testosterone [25–28]. As testosterone is known to be immune-suppressive [29, 30], this association between obesity related inflammation and lower levels of serum testosterone has previously been suggested to be caused by a reduction in testosterone’s immune-suppressive action [26]. However, observational studies are incapable of proving cause and effect, nor the mechanistic direction of such associations. Therefore we contend that the reduction in testosterone’s immune-suppressive effect is not the underlying cause of increased inflammation seen in obese men, but rather the reverse. Specifically, obesity triggers an inflammatory response that in turn impairs testicular function, and that this results in both a reduction in testosterone production and impaired spermatogenesis.

There is mounting evidence that obesity and a high in fat/calorie diet may provide a gut bacteria derived trigger for initiating inflammation throughout the body. The human gut contains nearly 2 kg, or 100 trillion (10^14^) bacteria, a population that outnumbers the body’s own eukaryotic cells by a 10-fold order of magnitude [31]. The bacterial density of the gut is relatively low in the proximal portion of the gastrointestinal tract (stomach, small intestine) due to the inhibitory effects of stomach acid and bile on bacterial growth, but reaches very large numbers (10^12^ CFU per gram faecal material) in the colon [31]. Many species of gut bacteria such as *bifidobacteria* and *lactobacillus* actually provide a beneficial symbiotic role to the human host, such as processing insoluble dietary fibre into short chain fatty acids that can be utilised by the host’s intestinal mucosa as an energy source, or the production of key vitamins such as Vitamin B12 and Vitamin K [31]. However, other bacterial species such as gram negative bacteria have clear pathogenic capacity, with the presence of such a huge number of bacteria within the body posing a significant potential threat to the host’s health.

The mucosal surface of the gastrointestinal tract covers an area equivalent to the size of a tennis court, allowing for the very efficient transfer of food and water from the gut lumen into the circulation. However, this also provides a large area of susceptibility for points of entry of harmful gut bacteria into the systemic circulation, where they can initiate activation of the body’s immune system and even overwhelming sepsis. Fortunately the trans-mucosal passage of gut bacteria is normally prevented by several mucosal barrier defence mechanisms, including the production of a thick mucus lining that repels bacteria from the intestinal surface, bactericidal antibodies and immune proteins, as well as tight junctions between the epithelial cells that ideally prevent passage of macro-molecules like endotoxin or intact bacteria between epithelial cells [32, 33].

Obesity, and a diet high in fat or calories that is typically consumed by obese individuals, has been reported to cause a breakdown in the normal mucosal barrier function, leading to the passage of gut bacteria into the systemic circulation, initiating a chronic state of inflammation [34, 35]. Gram negative bacteria, which comprise 70 % of the total bacterial load in the human gut [36], contain a potent immune stimulant in their cell wall referred to as lipopolysaccharide (LPS) or endotoxin. Animal experiments and human observational studies have shown that consumption of diets containing either high fat or high number of calories leads to significant changes in gut bacterial populations and increases in the circulating levels of plasma endotoxin [37, 38], implying a breakdown in gut mucosal wall integrity and the passage of gram negative bacteria into the systemic circulation. Interestingly, the magnitude of this “metabolic endotoxaemia” is reported to be more pronounced in mice placed on a high fat diet than an isocaloric high carbohydrate diet, suggesting that dietary fat is more efficient in transporting bacterial endotoxin from the gut lumen into the circulation, possibly mediated by transfer of endotoxin across the intestinal wall in lipid laden chylomicrons [34, 38]. Furthermore, a high fat diet is reported to unfavourably alter the gut microbial composition, leading to an increase in intestinal permeability due to disordered tight junction proteins (zonulin, occludin) [39], and a reduction in the colonic mucous barrier [40]. Confirming the importance of gut microbiome in facilitating endotoxaemia, the administration of antibiotics to obese mice or modification of their gut microbiome with prebiotic fibre, have both been reported to result in a decline in circulating plasma endotoxin levels [39, 41, 42].

Cross-sectional studies in humans have also reported an elevation in circulating levels of endotoxin [38, 43, 44], or indirect markers of endotoxin (LBP) exposure [45, 46], in obese individuals. Obesity has also been shown to be associated with changes in the human gut microbiome, with several investigators now reporting a reduction in the beneficial genus *bifidobacterium* in the faecal samples of obese individuals [47, 48]. Since *bifidobacterium* are known to metabolise dietary fibre, producing short chain fatty acids (SCFA) that “feed” the host intestinal mucosa, and enhance the production of mucus and maintain tight junction barrier function [49], it is likely that any reduction in *bifidobacterium* numbers due to obesity will result in a breakdown in intestinal barrier function and endotoxaemia. Furthermore, obese men have also been shown to have a more marked post-prandial endotoxaemic and inflammatory (IL-6) response to a standard meal containing 40 gm of fat than their age matched lean counterparts [34, 50]. As such, we propose that changes in the intestinal microbiome caused by obesity, and the associated “poor diet”, result in a breakdown in the mucosal barrier function of the gut (so called “leaky gut”), and that this results in the passage of gram negative bacteria into the circulation (metabolic endotoxaemia) which triggers a chronic state of inflammation that impairs testicular function.

### Endotoxin and impaired testicular function

Currently there is no experimental data supporting a direct link between endotoxin exposure in the male and impaired testosterone production or spermatogenesis. However, studies in women have confirmed an association between endotoxaemia and a reduction in the ovaries capacity to produce the female sex steroid hormone progesterone [46]. Furthermore, there is abundant animal evidence suggesting that endotoxin (LPS) does have the capacity to impair testicular function. Firstly, the experimental administration of LPS to rats, sheep, cattle and non-human primates has been shown to decrease the frequency and amplitude of LH pulses by suppressing both hypothalamic and anterior pituitary function [51], thereby reducing the pituitary drive for Leydig cells to produce testosterone. Secondly, animal studies have also confirmed that Leydig cells express the TLR4 for endotoxin [52], and that experimental administration of LPS directly inhibits Leydig cell production of testosterone [52–57]. The direct inhibition of androgen production by endotoxin is most likely mediated by a reduction in Leydig cell expression of steroidogenic acute regulatory (StAR) protein activity [58], a protein that plays a key role in the initial transfer of cholesterol into mitochondria where it is later converted into testosterone.

The activation status of testicular macrophages is also likely to play a role in testosterone production. Leydig cells and macrophages are normally in close physical contact within the testicular interstitium, and under normal conditions these macrophages play a key role in Leydig cell development as they provide essential growth and differentiation factors [58]. However, under immune-stimulatory conditions, as occurs with metabolic endotoxaemia, macrophages produce pro-inflammatory cytokines such as IL-1 and TNFα, plus reactive oxygen species (ROS), all known to reduce steroid hormone production by the adjacent Leydig cell [55, 57, 58]. Furthermore, Leydig cells themselves have been reported to produce inflammatory cytokines (IL-1β, TNFα and IL-6) when exposed to LPS [52], which would result in a further amplification of the neighbouring macrophages state of activation. Interestingly, dampening inflammation using TNFα blocking antibody therapy has been shown to normalise serum testosterone levels in spondylo-arthritis patients [59], highlighting the potential role for inflammation in decreasing testosterone production.

### Endotoxin and impaired sperm function

Obesity related endotoxaemia is likely to impair sperm production and function, both directly and indirectly. Firstly, high intra-testicular levels of testosterone are required for normal sperm production. Inadequate levels of testosterone disturbs Sertoli cell function, leading to retention and phagocytosis of mature spermatids [60] and impaired epididymal function, both potentially reducing sperm number and quality. Secondly, human sperm have been reported to express both the TLR4 [61] and the CD14 co-receptor for LPS [62], as well as directly responding to LPS exposure by increasing their production of IL-6 [63], initiating sperm apoptosis and a decline in sperm motility [61, 64–66]. Furthermore, as semen is known to contain both LPS and leukocytes [61], it is not surprising that endotoxin exposure would increase seminal leukocyte reactive oxygen species (ROS) production and result in sperm oxidative damage [67, 68]. Seminal plasma neopterin, a marker of macrophage activation status, has been reported to be increased in obese men [69], with seminal plasma neopterin also being positively correlate with sperm oxidative stress, DNA damage and apoptosis [69]. This finding, together with previous publications linking impaired sperm production with an increase in testicular macrophage density [70, 71], all support the concept that a trigger for inflammation such as metabolic endotoxaemia has the potential to impair spermatogenesis and sperm function.

## The evolutionary advantages associated with GELDING theory of endotoxin suppression of testicular function

In today’s environment of abundant high-calorie food and a resulting epidemic of obesity, it would appear that the GELDING concept of inflammatory mediated suppression of testicular function is maladaptive; with the resulting decline in testosterone production leading to a reduction in lean muscle mass and further predisposing to adiposity. However, outside of the context of the modern industrialised society, we believe that inflammatory suppression of testicular function may actually be an adaptive response, helping protect men from sepsis and preventing them from passing on their genes in times of sickness.

### Testosterone, immune responses and the immune-competence handicap

Testosterone is known to exert a suppressive effect on both humoral and cellular immune responses, and therefore appears to provide a natural anti-inflammatory advantage to men outside times of infection. Testosterone is reported to dampen the immuno-stimulatory activity of monocytes, macrophages, NK cells, T lymphocytes, as well as reducing antibody production by B lymphocytes [29]. As a result, autoimmune diseases such as systemic lupus erythematosus (SLE), rheumatoid arthritis, systemic sclerosis and myasthenia gravis are all significantly less common in men than women [30]. Conversely, men with androgen deficiency are at increased risk of autoimmune disease; with Sjogren syndrome, rheumatoid arthritis, autoimmune hypothroidism and SLE all being more common in hypogonadal men with Klinefelter’s Syndrome than their androgen replete counterparts [72]. Interestingly, many Klinefelter’s Syndrome patients with SLE experience a significant decline in their lupus activity once they commence androgen replacement therapy [73], highlighting the potent immune-suppressive actions of testosterone.

While testosterone may provide men with an autoimmune advantage, it also limits their capacity to fight infections, thereby resulting in increased rates of infectious morbidity and mortality compared to women [74, 75]. Folstad and Karter [76] were first to propose the concept of male “immunocompetence handicap”; a situation where males are required to balance the competing demands of high testosterone production for optimal reproductive performance (sperm production, development of male secondary sexual characteristics attractive to females and the maintenance of assertive territorial behaviour conducive to successful mating), with this “cost” of high testosterone being diminished immune capacity and susceptibility to infection. For example, dominant “alpha” male reindeers, baboons and chimpanzees, known to possess both the highest levels of testosterone and reproductive performance, also have been reported to have the greatest parasitic infective load compared with non-dominant or castrated males [76–78]. Interestingly, castration of dominant males has the ability to reduce their susceptibility to these types of parasitic infections [79], while experimental treatment with high dose testosterone increases the intensity of parasitic infection and resultant mortality [80, 81].

What constitutes the optimal adaptive balance between high testosterone levels and reproductive performance, versus lower testosterone levels and resistance to infection, depends on the longevity of the animal and its social behaviour. Australian marsupials such as the dasyurid (quoll) have evolved a semelparious, or so called “big-bang” suicidal reproductive behaviour, where a male engages in a single frenzied mating season in their entire life fuelled by high testosterone levels, but then dies shortly after mating from infection brought about by a total collapse in their immune system [82–84]. In an environment with limited food resources, and where the male plays no active role in the upkeep of his progeny, this type of semelparous reproductive strategy may actually be adaptive since it enables him to pass on his genes, while not competing for limited resources with his offspring. However, the optimal balance between high testosterone and reproductive performance and immunity is likely to be very different for humans. Firstly, men are expected to play an active role in supporting their children over a number of years, with the death of a father having a major detrimental effect on their children’s welfare. As such, high immune competence and longevity are of paramount importance to men and their families. Secondly, for most part humans reproduce in a monogamous setting, where males do not need to compete with other males for an opportunity to “mate”, unlike the animal world where males require high testosterone to develop body strength and aggressive behaviour in order to defend their territory and attract a female mate. Therefore, the human male only requires sufficient testosterone to maintain normal spermatogenesis, but not supra-physiological levels that will unnecessarily suppress his immune system and potentially compromise his survival.

The ability for an infection to reduce testosterone production, thereby removing this hormonal brake on infection fighting capacity, is supported by the available literature. Several animal studies using experimental administration of endotoxin (LPS) as a surrogate for sepsis has shown that endotoxin initiated inflammation is a powerful inhibitor of testosterone production [52–57]. Similar studies in men have reported that endotoxin does suppress the production of the adrenal androgen DHEA [85], although no study to date has reported the effect of experimental administration of endotoxin on testosterone levels. However, a prospective study of 28 men has reported a significant reduction in serum testosterone and an elevation in estrogen during times of severe sepsis [86], a pattern identical to what we have proposed to occur in obese males as a result of metabolic endotoxaemia. While the magnitude of endotoxaemia in sepsis is approximately 10-50 fold higher than that seen in obesity [35], we still believe that it is reasonable to conclude that chronic exposure to low grade endotoxinaemia may interfere with testosterone production, in support of the GELDING theory.

### Testosterone, infection and “reproductive fitness”

Male fertility has been shown to transiently decline during times of infection, with a significant reduction in sperm count, motility, morphology and DNA integrity [87–90], plus a reduction in sperm fertilising capacity all being reported [91]. Previously it has been postulated that these reductions in sperm quality were due to an elevation in core body temperature (fever) that commonly occurs during infection, since spermatogenesis is optimal at 35 °C [24]. However, chronic low grade infections with Hepatitis B and HIV have also been reported to cause a reduction in sperm quality, without any change in body temperature [92, 93]. Interestingly, around 25 % of young to middle-aged men chronically infected with HIV have hypogonadism and androgen deficiency [94], with the reduction in their sperm quality being directly proportional to the severity of their infectious load (CD4+ count) [93]. As such, it appears highly probable that chronic infection and its associated inflammatory response are capable of impairing testicular function and producing a drop in sperm quality and testosterone production. While we acknowledge that viral and parasitic infections do not result in exposure of the host to LPS, an immune stimulant exclusively found in gram negative bacteria, these observations do support the GELDING hypothesis as they provide evidence that an inflammatory stimulus (viral, parasitic or bacterial exposure) can result in impaired testicular function.

From an evolutionary perspective, it is obvious that a male who is unhealthy due to infection should ideally not be capable of siring offspring. Firstly, sickness may signify a poor genetic endowment (propensity to illness), a characteristic that is best not passed on to the next generation. Secondly, since infection has been linked with a reduction in sperm DNA integrity [88, 90], and poor sperm DNA quality has been linked with an increased risk of miscarriage and illness in the resultant offspring [95], a block in the capacity of unhealthy males to reproduce makes perfect evolutionary “Darwinian” sense. The decline in sperm quality with infection provides the ideal biological roadblock preventing such conceptions.

A second roadblock to unhealthy males reproducing is the observed reduction in libido and social withdrawal. The experimental replication of infection through administration of endotoxin (LPS) to men has been reported to produce depression, anxiety, fatigue and a sense of social disconnection [96], so called “sickness behaviour”. Of course all of these psychological symptoms are likely to significantly reduce the probability of a sexual encounter and successful reproduction. While no study to date has directly analysed the link between the administration of endotoxin to men, the onset of sickness behaviour and changes in serum testosterone, it is highly probable that these symptoms are at least in part due to an acute suppression in testosterone production. Firstly, the sickness behaviours associated with experimental administration of endotoxin (decreased mood, fatigue, social disconnection, anhedonia) very closely resemble the psychological symptoms associated with androgen deficiency [3, 96]. Secondly, animal models of sickness behaviour using experimental administration of endotoxin report that male behaviour can be normalised by co-administration of testosterone therapy [97], highlighting the role of androgen deficiency in endotoxin mediated sickness behaviours. In the setting of infection, a decline in activity and social interest is adaptive since it allows the body to rest and recover, while also reducing the chance of spreading the infection to others. However, in the setting of obesity related metabolic endotoxaemia the chronic adoption of sickness behaviour (altered mood, poor motivation, and social isolation) is clearly maladaptive. As such, more research is needed to investigate the potential links between obesity, metabolic endotoxaemia, and impaired testicular function plus potential treatments for this significant malady.

## Novel therapies to combat obesity related impairment of gonadal function

### Current treatments for impaired reproductive function in obese men

Current therapies for male hypogonadism primarily address the symptoms of androgen deficiency by initiating testosterone replacement therapy, rather than treating the underlying pathology (testicular inflammation). While androgen therapy can be very effective at reversing the psychological and physical symptoms of androgen deficiency [3], it also inhibits spermatogenesis, often causing azoospermia [98]. Therefore traditional androgen replacement therapy is unable to treat both aspects of obesity related hypogonadism -low testosterone and subfertility.

Weight loss through diet and exercise is reported to result in a significant improvement in testosterone levels [8–10], however sustained weight loss over a long duration is accomplished by only a minority of obese men. Bariatric surgical procedures are known to be more effective in producing long term weight loss than diet and exercise alone [99], and several investigators have now reported improvements in obese men’s sex hormone status [100–103], plus semen quality [104] with this type of surgical approach. Furthermore, one study did report a significant decline in inflammation (serum CRP) and a corresponding increase in testosterone following weight loss surgery [100], but unfortunately failed to analyse the correlation between these two outcomes, nor did they measure changes in endotoxin exposure. Finally it should be recognised that bariatric surgery is not without its risks, and therefore other less invasive alternative therapies are still needed.

### Modification of the intestinal microbiome in order to treat obesity related hypogonadism

According to the GELDING theory of hypogonadism, the key to effective treatment of androgen deficiency and impaired fertility in obese men is to improve the barrier function of the intestine, thereby preventing trans-migration of bacteria from the bowel lumen into the systemic circulation through a leaky gut wall. The resultant reduction in metabolic endotoxaemia would improve testicular function both directly and indirectly through increased pituitary LH drive.

Therapies directed at changing the resident bowel flora (microbiome) have recently gained interest as potential treatments for improving human health. This is achieved by either administration of probiotic bacterial supplements, or prebiotic supplements that nourish beneficial bowel bacteria. A probiotic is defined as “a live microorganism which when administered in adequate amounts confers a health benefit to the host” [105]. Probiotics are often referred to as “good” or “beneficial bacteria” since they limit the growth of “bad” bacteria like endotoxin containing gram negative bacteria. The two most commonly used probiotic organisms are *bifidobacterium* and *lactobacillus*, since both of these bacteria are non-pathogenic and known to enhance gut health [105].

Probiotic bacteria may help prevent metabolic endotoxaemia through two distinct mechanistic pathways. Firstly these beneficial bacteria reduce the intestinal load of potentially harmful gram negative bacteria by inhibiting the growth of these bacteria through lowering colonic pH, competing for nutrients and enhancing the secretion of antibacterial immunoglobulins and bactericidal compounds by the intestinal mucosa [32, 33]. Secondly, probiotic bacteria are capable of producing short chain fatty acids (SCFA) which “feed” the adjacent intestinal wall, increasing the health and barrier function of the mucosal surface [49]. SCFA have been reported to increase the production of mucous by goblet cells within the colonic wall, thereby providing a physical barrier that reduces contact with bacteria in the gut lumen, minimising the potential for trans-migration of these bacteria into the systemic circulation. In addition, SCFA derived from beneficial bacteria are known to enhance the production of epithelial tight junction proteins that prevent passage of macromolecules such as endotoxin between intestinal epithelial cells [49].

Prebiotics are best described as a selective food source for beneficial “good” bacteria, or as “a selectively fermentable ingredient that allows specific changes, both in the composition and/or activity in the gastrointestinal microflora that confers benefits upon host wellbeing and health” [105]. In order for a substance to be considered prebiotic it must not be digestible by the host, instead being delivered relatively intact to the colonic lumen where it may act as nutrients for beneficial bacteria. Secondly, the prebiotic substance must support the growth and function of beneficial bacteria such as *bifidobacterium* and *lactobacillus*, while not facilitating the growth of non-beneficial “bad” bacteria. Currently the long chain fructo-oligosaccharide inulin, present in wheat, onion, bananas, garlic, asparagus and artichoke, is the most commonly ingested prebiotic. However, as these foods contain only relatively small amounts of inulin, most prebiotic supplements contain inulin soluble fibre derived from the processing of chicory root. Frequently, the most effective manner of improving the gut microbiome is the combined application of probiotics with prebiotic- so called symbiotic therapy [105].

Currently there is significant animal experimental evidence suggesting that the use of probiotic therapy can improve the intestinal wall barrier function and result in a reduction in levels of endotoxaemia [106]. Administration of the probiotic bacteria *akkermansia mucinophilia* to mice has been reported to enhance the colon’s mucous barrier and result in a drop in systemic endotoxin levels [40]. Similarly, the use of lactobacillus probiotic therapy in mice has been reported to increase the intestines production of barrier function occludin and claudin-1 tight junction proteins [107], and decrease gut permeability [108], thereby resulting in a reduction in systemic endotoxin exposure [107, 109]. Furthermore, a recent randomised controlled trial reported a significant reduction in inflammation (high sensitivity CRP levels) in obese adults given a 3 month probiotic supplement containing *bifidobacterium* [110], although it is uncertain if this reduction was mediated by a decline in metabolic endotoxaemia, or whether these changes translated into improvements in serum testosterone.

Animal studies have now provided the first evidence supporting the ability of probiotic supplements to boost testicular function in obese subjects. In one study, the consumption of a probiotic mixture containing *lactobacillus* and other probiotic bacteria by rats fed a high fat diet was reported to be able to prevent sperm oxidative stress and the associated reduction in sperm quality [111]. Similarly, another group noted that male mice fed a diet containing the probiotic bacteria *lactobacillus reuteri* had larger testicles, greater Leydig cell density and higher serum testosterone and increased spermatogenesis compared to controls [112]. Interestingly, in order to test whether the beneficial effects of the *L. reuteri* probiotic were associated with an anti-inflammatory mechanism, these investigators administered antibodies that blocked the action of the pro-inflammatory cytokine IL-17. Those mice depleted of IL-17 activity were found to have greater testicular volume and higher Leydig and germ cell density compared to sham treated controls, similar to what was also observed in the *L. reuteri* treated males, thereby supporting the conclusion that *L. reuteri* probiotic most probably prevents aged related decline in testicular function by dampening inflammation.

### Future research directions

Going forward, several studies need to be conducted in order to support or refute the GELDING hypothesis. Firstly, we personally anticipate conducting observational studies examining the relationship between serum testosterone and estrogen, semen quality, various measures of adiposity (BMI, waist circumference, percentage body fat) and levels of endotoxin exposure. We anticipate that endotoxin exposure will be negatively associated with both serum testosterone and sperm quality. In addition, it would also be interesting to correlate direct measures of intestinal permeability, such as sugar absorption tests [113], with changes in testicular function. Secondly, as observational studies can never prove causation themselves, we would like to see if the experimental administration of endotoxin (LPS) to healthy men does result in changes in serum testosterone, estrogen and sperm production. These types of “low dose endotoxaemia” studies have already been conducted by many investigators in the fields of cardiovascular and behavioural science [85, 114], with endotoxin exposure being well tolerated by participants, yet no study to date has measured the impact of endotoxin exposure on male reproductive function. Interestingly, a recent study which randomised men to an infusion of the pro-inflammatory cytokine IL-2 or saline placebo, did confirm that initiation of a systemic state of inflammation did result in a significant reduction in serum testosterone [115]. Furthermore, if our GELDING theory of obesity related hypogonadism is supported by the experiments outlined above, then we believe that it would be prudent to commence trials examining ways in which modification of gut permeability may improve testicular function. Here the use of pre and probiotic supplements to modify the gut microbiome and enhance intestinal barrier function appears to be an obvious initial therapeutic choice for augmenting testicular function in obese men. Any therapy that can reverse hypogonadism in obese men is likely to significantly improve their quality of life (increased energy and sexual function, improved mood and motivation), but also may produce weight loss by increasing activity and muscle mass, leading to a reduction in the incidence of cardiovascular disease and other obesity related medical conditions. Finally, as endotoxin-related inflammation is associated with insulin resistance, type 2 diabetes mellitus and cardio-vascular disease [37, 41–45, 114], new therapeutic approaches that improve gut barrier function producing a reduction in endotoxaemia and inflammation may also reduce these types of metabolic diseases [35, 110]. These are certainly worthwhile research goals to investigate in the future.

As with most diseases, we acknowledge that obesity related male hypogonadism is likely to have several underlying triggers, with metabolic endotoxaemia playing one significant role in the disease patho-physiology. It is almost certain that other processes unrelated to endotoxin or inflammation, such as adipose tissues conversion of testosterone to estrogen and the resulting negative feedback on the HP axis, also play significant roles in impairing testicular function. The purpose of this paper is to inform the scientific community of the role that gut derived endotoxin has as one of many potential causes of obesity related hypogonadism.

## Conclusions

While we acknowledge that currently there is no human data directly linking endotoxin exposure to impaired testicular function, we still believe that there is considerable circumstantial evidence supporting such a theory. Firstly, obesity and a high fat diet have both been conclusively linked with changes in gut microbiota, increased intestinal permeability and the resultant leakage of bacterial endotoxin from the gut lumen into the systemic circulation (metabolic endotoxaemia) [38, 43–46]. Secondly, animal studies have clearly shown that exposure to endotoxin does result in a reduction in testosterone production, both indirectly (impaired pituitary LH drive), and through direct inhibition of Leydig cell function [51–57]. While similar studies have not yet been conducted in men, it has been reported that serum testosterone levels do fall during times of infectious endotoxin exposure [86], as anticipated by the GELDING theory. Furthermore, multiple large observational studies have now linked increased levels of inflammation (raised CRP and WCC) with lower serum testosterone [25–28].

The GELDING theory is entirely novel in that for the first time it provides a clue to what may be initiating inflammation and impairing testicular function in obese men- gut derived endotoxin. If proven correct, the GELDING theory opens up a whole new scope for treatment of the hypogonadal male through modification of his gut microbiome and intestinal permeability. For example, obesity related hypogonadism becomes more common with increasing age, causing significant physical and psychological impairment. However, modification of the gut microbiome using probiotics has already been reported to reverse this age-related hypogonadism in rodents [112], raising exciting therapeutic potential for older men.

Finally, the GELDING theory poses the interesting and important evolutionary concept that endotoxin related suppression of testicular function may originally have been an adaptive response in times of sepsis (removing testosterone mediated immunosuppression, preventing sick males reproducing). However, such a response in today’s world of food abundance is now more commonly maladaptive, where “non-infectious” metabolic endotoxaemia related androgen deficiency significantly reduces obese men’s fertility and quality of life.

## Abbreviations

AMH, antimullerian hormone; BMI, body mass index; CFU, colony forming units; CRP, C-reactive protein; DHEA, dehydroepiandrosterone; DNA, deoxyribonucleic acid; GELDING, gut endotoxin leading to a decline in gonadal function; HIV, human immunodeficiency virus; HP, hypothalamic-pituitary; IL-1, interleukin 1; IL-6, interleukin 6; INSL3, insulin like growth factor 3; LBP, lipopolysaccharide binding protein; LH, luteinizing hormone; LPS, lipopolysaccharide; ROS, reactive oxygen species; SCFA, short chain fatty acids; SLE, systemic lupus erythematous; StAR, steroidogenic acute regulatory protein; TLR4, toll-like receptor 4; TNFa, tumour necrosis factor alpha; WCC, white cell count
